# Supplementary material for: Identification of CO2 adsorption sites on MgO nanosheets by solid-state nuclear magnetic resonance spectroscopy
Source: Nat Commun. 2022 Feb 4;13:707. doi: 10.1038/s41467-022-28405-6 (PMC8817041; doi:10.1038/s41467-022-28405-6)
Supplement: Supplementary file 1 — Supplementary Information [file 41467_2022_28405_MOESM1_ESM.pdf]

Supplementary Information

for

Identification of CO_2 Adsorption Sites on MgO Nanosheets by Solid-state Nuclear Magnetic Resonance Spectroscopy

Jia-Huan Du[1], Lu Chen[2], Bing Zhang[3], Kuizhi Chen[4], Meng Wang[5], Yang Wang[1],

Ivan Hung[4], Zhehong Gan[4], Xin-Ping Wu[2], Xue-Qing Gong[2], and Luming Peng[1,*]

[1]Key Laboratory of Mesoscopic Chemistry of Ministry of Education, School of Chemistry and Chemical Engineering, Nanjing University, Nanjing 210023, China.

[2]Key Laboratory for Advanced Materials, Centre for Computational Chemistry and Research Institute of Industrial Catalysis, East China University of Science and Technology, 130 Meilong Road, Shanghai 200237, China.

[3]Lam Research Corporation, Fremont, California 94538, USA.

[4]National High Magnetic Field Laboratory, 1800 East Paul Dirac Drive, Tallahassee, FL 32310–3706, USA.

[5]College of Chemistry and Molecular Engineering (CCME), Peking University, Beijing 100871, China.

* E-mail: luming@nju.edu.cn (Luming Peng)

Phone: +86-151-0517-1931

Table of Contents for Supplementary Information

Supplementary Methods

DFT calculations of MgO(111) model..3

Preparations of Au/MgO..5

Supplementary Figures

XRD and HRTEM characterization...6

^{17}O NMR spectra of MgO nanosheets enriched with $^{17}O_2$ at different temperature…8

Longitudinal relaxation time measurements..10

^{13}C MAS NMR spectra of MgO nanosheets after $^{13}CO_2$ adsorption...........................11

Adsorption isotherms...12

^{1}H and ^{13}C MAS NMR spectra of MgO nanosheets...13

^{17}O MAS NMR spectra of MgO nanosheets with a larger frequency range...............15

^{17}O NMR spectra of MgO nanosheets after exposing to $H_2{}^{17}O$.................................16

^{17}O-^{1}H REDOR NMR data 18

Schematic representation of the surface structure of MgO(111) nanosheets.............20

UV-Vis characterization...21

DFT calculations of CO_2 adsorption on MgO(111) 22

^{17}O NMR spectra of Au/MgO...27

Supplementary References...28

Supplementary Methods

DFT Calculations.

Spin-polarized DFT calculations were performed using the *Vienna Ab initio Simulation Package* (*VASP*)[1,2] with the projector augmented-wave (PAW)[3] method. The Perdew−Burke−Ernzerhof (PBE) functional was used to deal with electronic exchange and correlation[4]. Wave functions were expanded in plane-waves with a cutoff energy of 450 eV. The H (1*s*), C(2*s*, 2*p*), O (2*s*, 2*p*), and Ce (4*f*, 5*s*, 5*p*, 5*d*, 6*s*) shells were treated as valence electrons. Geometry optimizations were finished until the Hellman-Feynman force on each ion was less than 0.05 eV/Å. The 5×5×5 and 1×1×1 *k*-point meshes were used for the Brillouin-zone integrations in bulk and surface calculations, respectively. The calculated lattice parameter of magnesia (4.23 Å) was in good agreement with the experimental value (4.24 Å)[5].

The polar MgO(111) was simulated by a 12-layer-slab which contains six layers of O and six layers of Mg (see Supplementary Fig. 15). The MgO(111) was extended at a (4 × 4) surface cell (area: 12 Å × 12 Å × sin 60° = 125 $Å^2$). The slab model contains a large vacuum gap (> 15 Å) along the z axis in order to avoid artificial surface-surface interactions. During geometry optimizations, the four middle atomic layers, including two O layers and two Mg layers, were kept fixed, while the other atomic layers were free to relax. The oxygen-terminated MgO(111) surface was used to study CO_2 adsorptions. The hydrogen coverage of the hydroxylated MgO(111) is 1/4 ML, which matches well with the experimentally observed result of 4 H atoms nm^{-2}.

The free energies of CO_2 adsorption (G_{ads}) were calculated as follows:

$$G_{\mathrm{ads}} = G_{\mathrm{CO_2/surf}} - G_{\mathrm{surf}} - \mu[\mathrm{CO_2}](p,T) \qquad (1)$$

where $G_{\mathrm{CO_2/surf}}$ and G_{surf} are the Gibbs free energies of the adsorption complex and the clean or hydroxylated MgO(111) surface, respectively, and $\mu[\mathrm{CO_2}](p,T)$ is the chemical potential of a gas-phase CO_2 molecule at given partial pressure (p = 170 mbar) and temperature (T = 313.15 K). Since the Gibbs free energies of the solid states can be well approximated by the corresponding DFT total energies, supplementary equation (1) can be rewritten as follows:

$$G_{\mathrm{ads}} = E_{\mathrm{CO_2/surf}} - E_{\mathrm{surf}} - \mu[\mathrm{CO_2}](p,T) \qquad (2)$$

where $E_{\mathrm{CO_2/surf}}$ and E_{surf} are the DFT total energies of the adsorption complex and the clean or hydroxylated MgO(111) surface, respectively.

We assumed that the surfaces are in thermodynamic equilibrium with gas phase. So, $\mu[\mathrm{CO_2}](p,T)$ can be calculated as following:

$$\mu[\mathrm{CO_2}](p,T) = E_{\mathrm{CO_2}} + \Delta\mu[\mathrm{CO_2}](p,T) = E_{\mathrm{CO_2}} + \Big\{H[\mathrm{CO_2}](p^0,T) - H[\mathrm{CO_2}](p^0,0K) - TS[\mathrm{CO_2}](p^0,T) + K_B T \ln\frac{p}{p^0}\Big\} \qquad (3)$$

where $E_{\mathrm{CO_2}}$ is the DFT total energy of a gas-phase CO_2 molecule, and enthalpy (H) and entropy (S) terms were taken from literature[6].

Therefore, G_{ads} can be given by:

$$G_{\mathrm{ads}} = E_{\mathrm{CO_2/surf}} - E_{\mathrm{surf}} - E_{\mathrm{CO_2}} - \Big\{H[\mathrm{CO_2}](p^0,T) - H[\mathrm{CO_2}](p^0,0K) - TS[\mathrm{CO_2}](p^0,T) + K_B T \ln\frac{p}{p^0}\Big\} \qquad (4)$$

Note that the vibration contributions and the pV term of solid components were neglected.

Preparations of Au/MgO.

200 mg MgO nanosheets (NS-1073) powder was suspended in 10 mL 1.2 mM $HAuCl_4$ in ethanol and stirred for 24 h. The mixture was then centrifuged and resuspended in ethanol for three times, and finally dried at 393 K overnight. The obtained powder is denoted as Au/MgO, which has 1.1 wt% Au according to the ICP result. Au/MgO sample was labeled with ^{17}O by using the same procedure as NS-1073.

Supplementary Figures

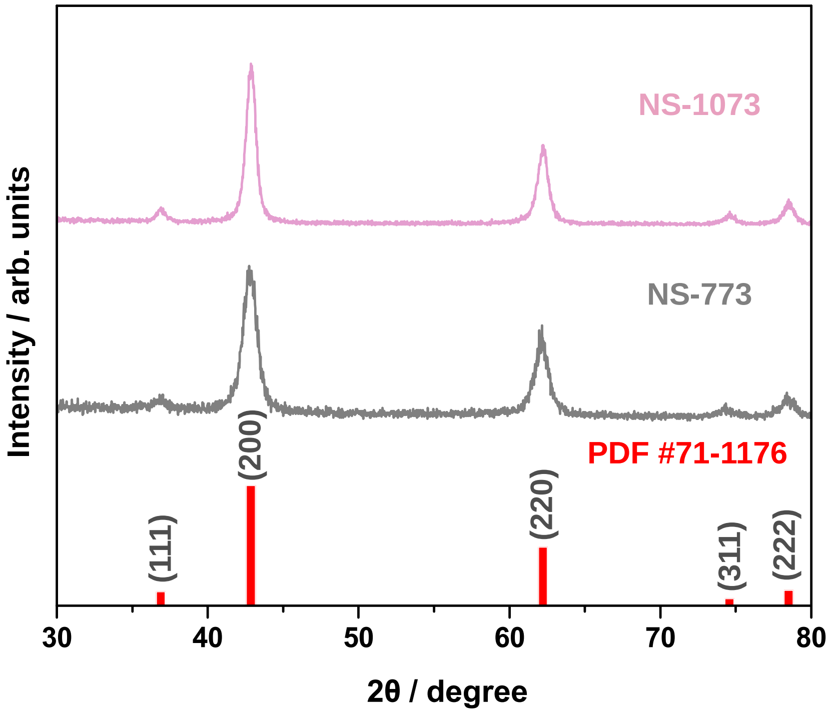

Supplementary Fig. 1. The XRD patterns of MgO nanomaterials heated at different temperatures in comparison to the diffraction peaks of MgO (PDF No. 71-1176). The average crystal sizes of MgO nanosheets calculated based on the XRD data using the Debye-Scherrer equation, are approx. 7 and 10 nm for the samples calcined at 773 and 1073 K, respectively.

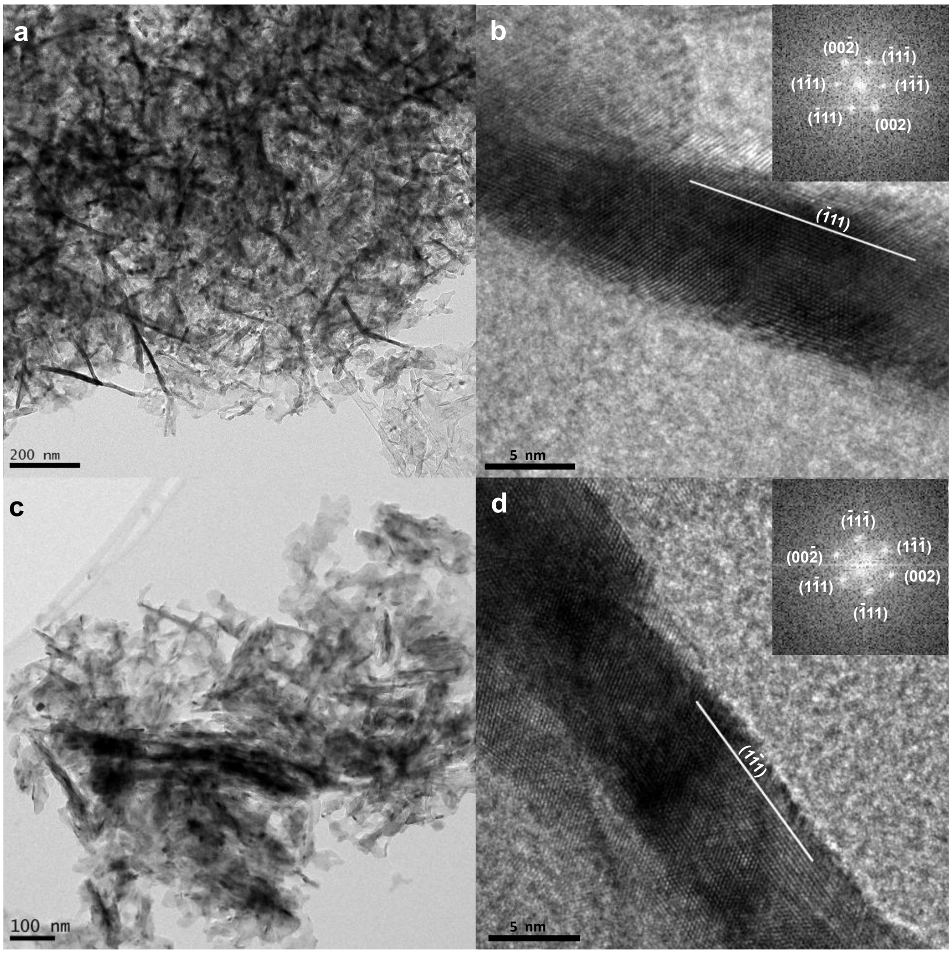

Supplementary Fig. 2. HRTEM images of MgO(111) nanosheets heated at 1073 K (a,b) and 773 K (c,d). Corresponding fast Fourier transform images are shown as inset in (b) and (d).

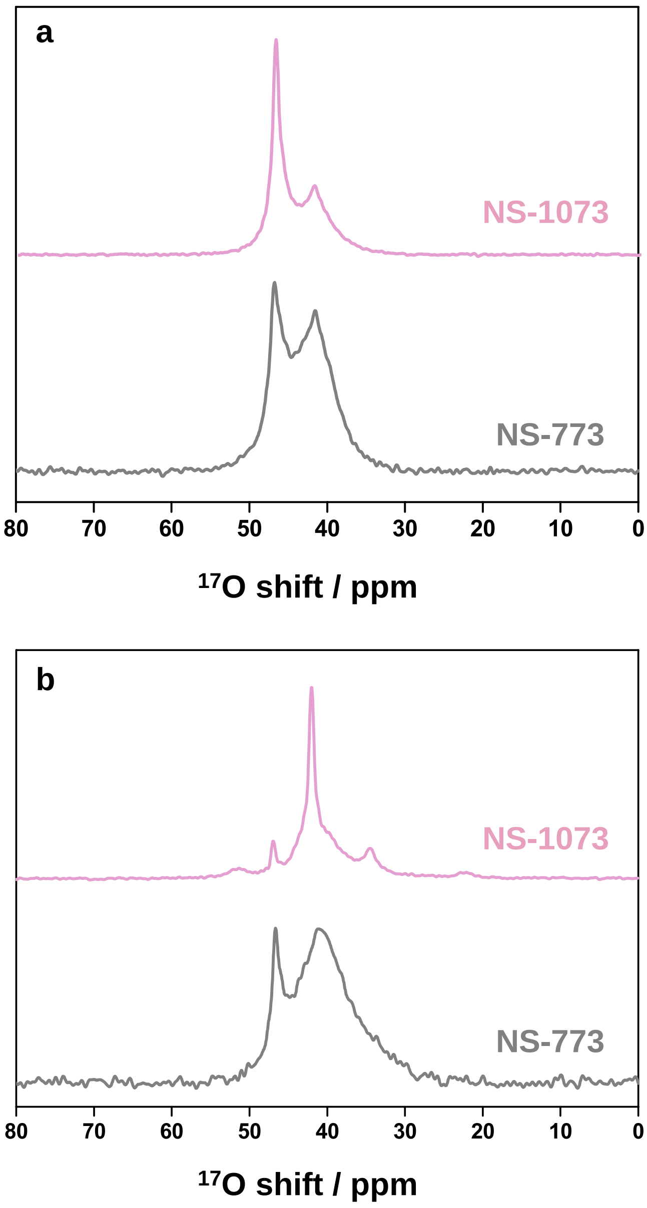

Supplementary Fig. 3. ^{17}O NMR spectra of NS-773 and NS-1073 enriched with $^{17}O_2$ at 773 (a) and 623 K (b). External magnetic field: 9.4 T; MAS rate: 20 kHz; recycle delay: 5 s. The peak at 47 ppm, arising from the oxygen ions in the bulk part of nanosheets, contribute more to the total intensity for both NS-773 and NS-1073 enriched at 773 K, compared to the data of corresponding samples enriched at 623 K. The spectra are plotted such that the highest peak in each spectrum are at the same

height and the relative intensities of different peaks can be conveniently compared. For the two spectra collected for NS-1073 (enriched at different temperature), since the enrichment temperatures (623 and 773 K) are lower than the thermal treatment temperature of 1073 K, the surface should be the same. Therefore, difference in the relative intensity of the signal from the bulk (or the surface) can be attributed to the diffusion of oxygen to the bulk. A higher enrichment temperature leads to more significant diffusion of ^{17}O into the bulk, and thus a stronger signal at 47 ppm. Similar situation is found for the two spectra acquired for NS-773.

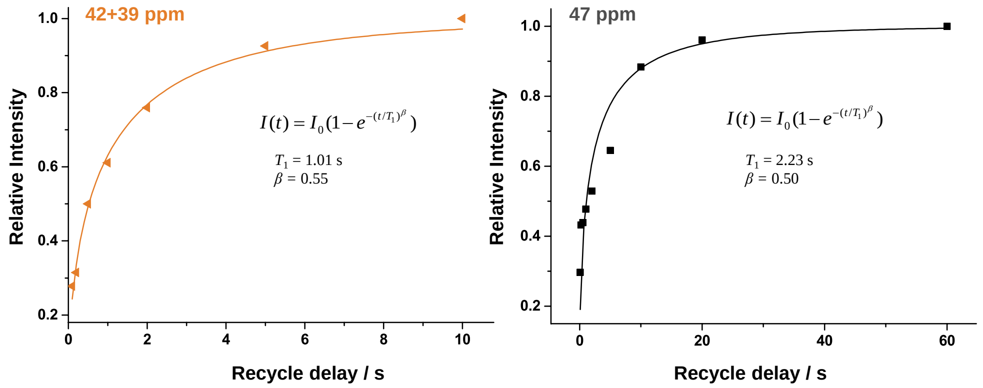

Supplementary Fig. 4. Spectral intensities as a function of recycle delay used for NS-773, along with fitted exponential curve, $I(t)=I_0(1-e^{-(t/T_1)^\beta})$, where $I(t)$ and I_0 are the signal intensities at recycle delay t and at equilibrium respectively. Clearly, the overlapping peak at 42 and 39 ppm is associated with a shorter longitudinal relaxation time (T_1) than the peak at 47 ppm. External magnetic field: 9.4 T; MAS rate: 16 kHz.

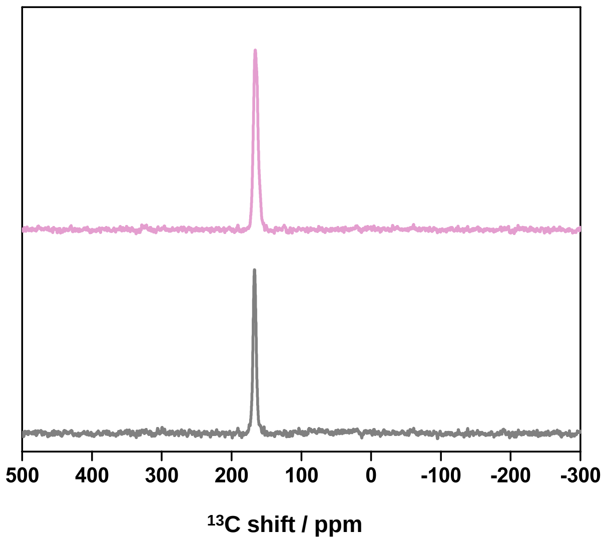

Supplementary Fig. 5. ^{13}C single pulse MAS NMR spectra of NS-773 and NS-1073 after $^{13}CO_2$ chemisorption with a larger frequency range at 9.4 T. MAS rate: 16 kHz; recycle delay: 40 s.

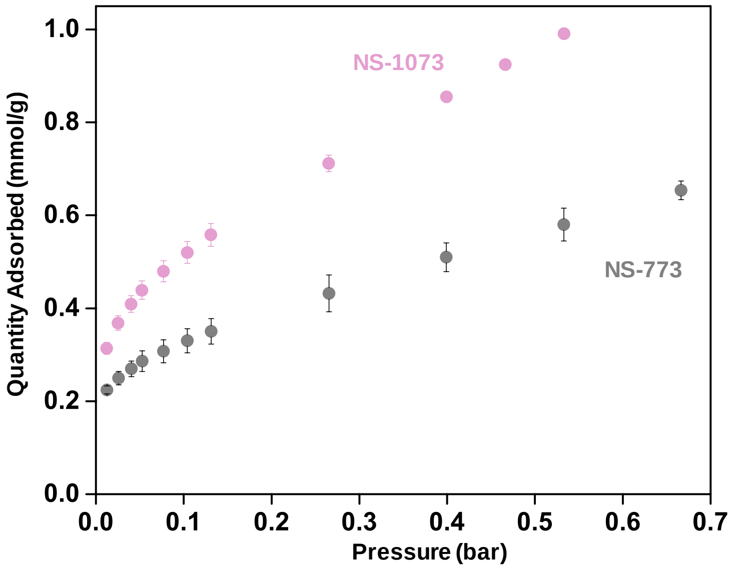

Supplementary Fig. 6. CO_2 adsorption isotherms ($T = 313$ K) on NS-773 and NS-1073.

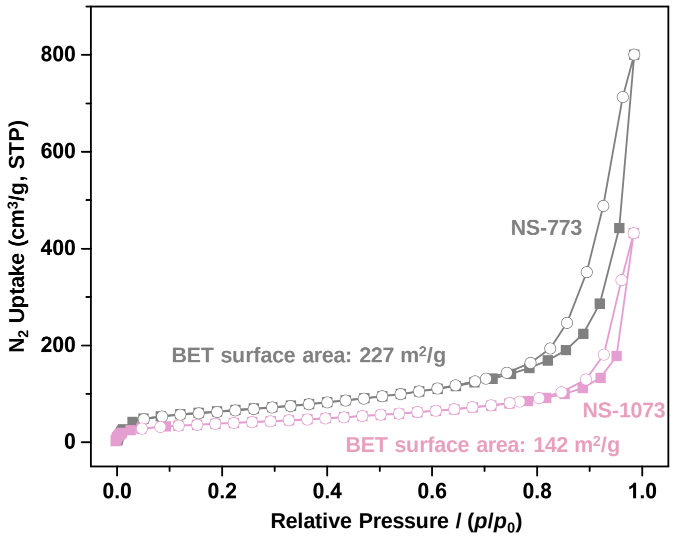

Supplementary Fig. 7. Nitrogen adsorption-desorption isotherms of NS-773 and NS-1073.

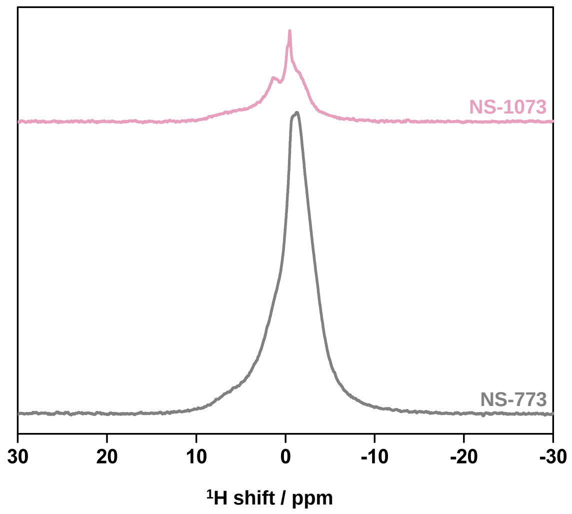

Supplementary Fig. 8. ^1H MAS NMR spectra of NS-773 and NS-1073. External magnetic field: 9.4 T; MAS rate: 16 kHz; recycle delay: 5 s.

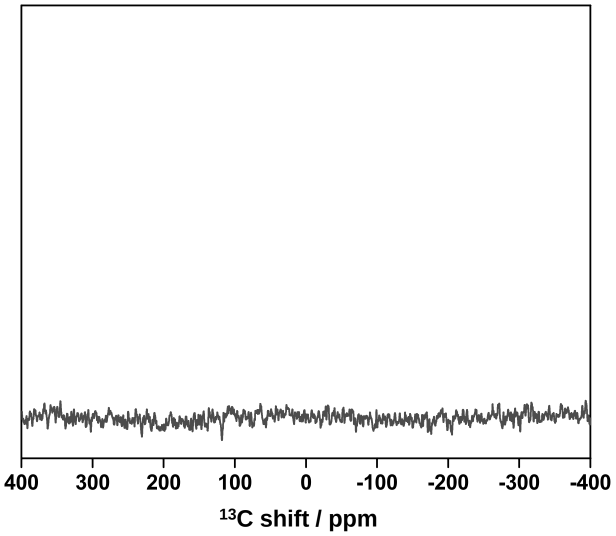

Supplementary Fig. 9. ^{13}C single pulse MAS NMR spectra of NS-773 at 9.4 T. MAS rate: 15 kHz; recycle delay: 20 s. A total of 200 accumulations were collected.

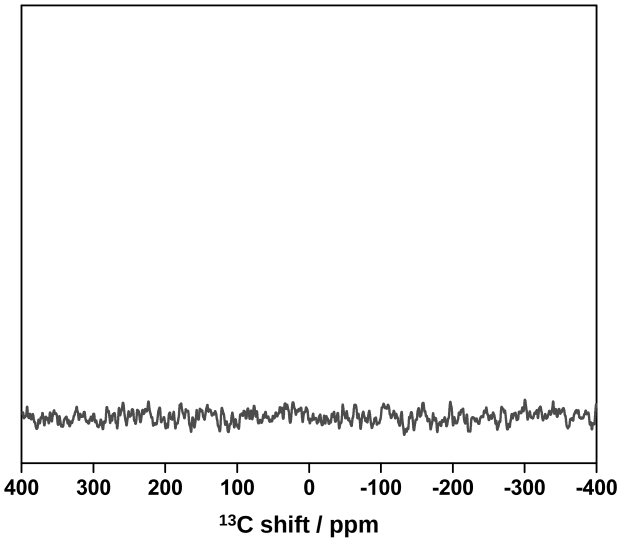

Supplementary Fig. 10. $^{1}H\rightarrow^{13}C$ cross polarization (CP) MAS NMR spectra of NS-773 at 9.4 T. MAS rate: 8 kHz; recycle delay: 2 s. A total of 4000 accumulations were collected.

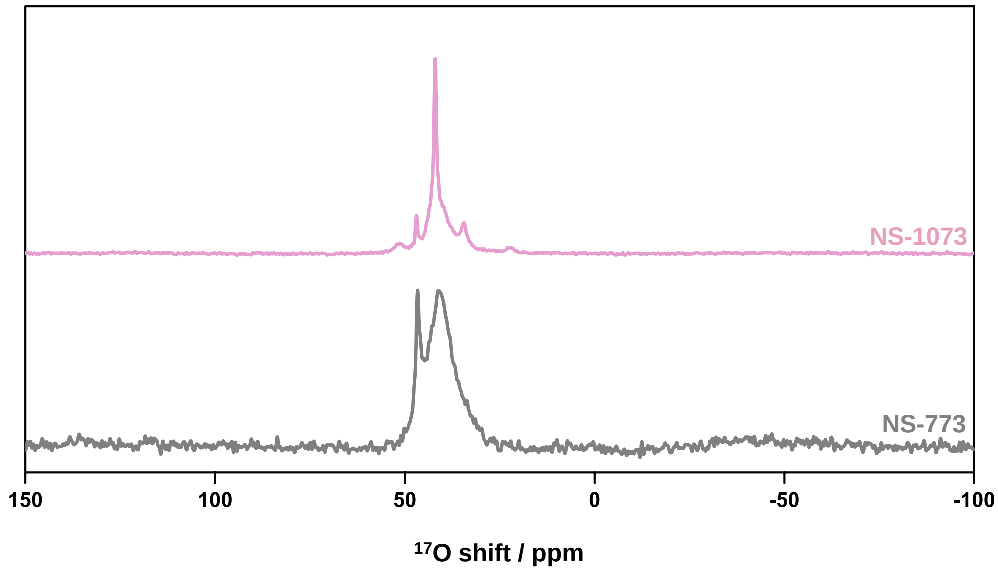

Supplementary Fig. 11. ^{17}O NMR spectra of NS-773 and NS-1073 with a larger frequency range. External magnetic field: 9.4 T; MAS rate: 20 kHz; recycle delay: 5 s.

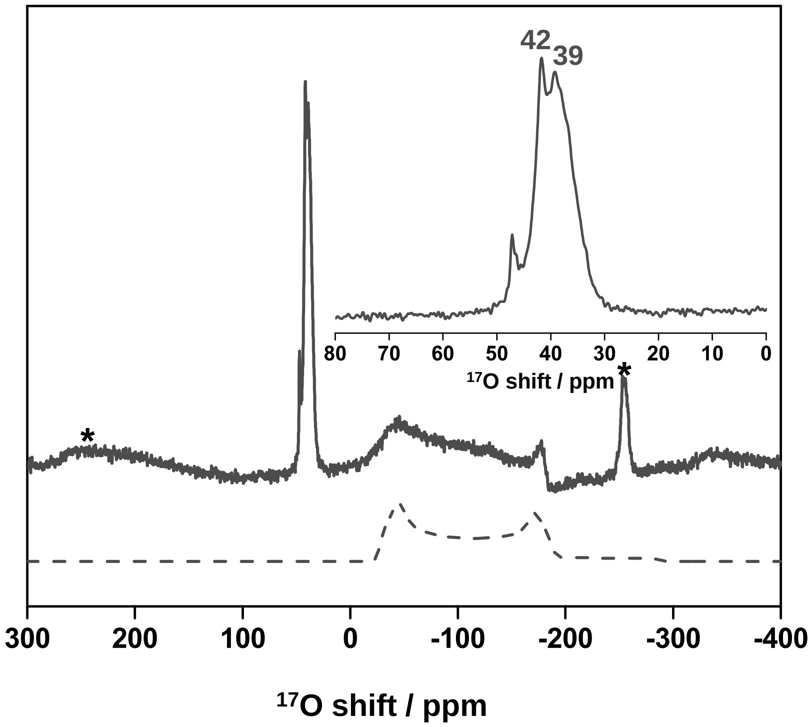

Supplementary Fig. 12. ^{17}O NMR spectrum of ^{17}O enriched NS-773 exposed to $H_2{}^{17}O$ (90%, 4 mbar). External magnetic field: 9.4 T; MAS rate: 16 kHz; recycle delay: 0.5 s. The inset in the upper right corner shows the enlarged region from 80 to 0 ppm. Asterisks denote spinning sidebands. A new and broad peak can be observed at 0 to -200 ppm, which can be simulated with the following NMR parameters (simulation: dashed line, C_Q = 7.6 MHz, η = 0.1, δ_{iso} = 5 ppm). This signal can be attributed to surface hydroxyls. The peaks at 47 and 42 to 39 ppm are signals from oxygen ions in the bulk and surface sites, respectively.

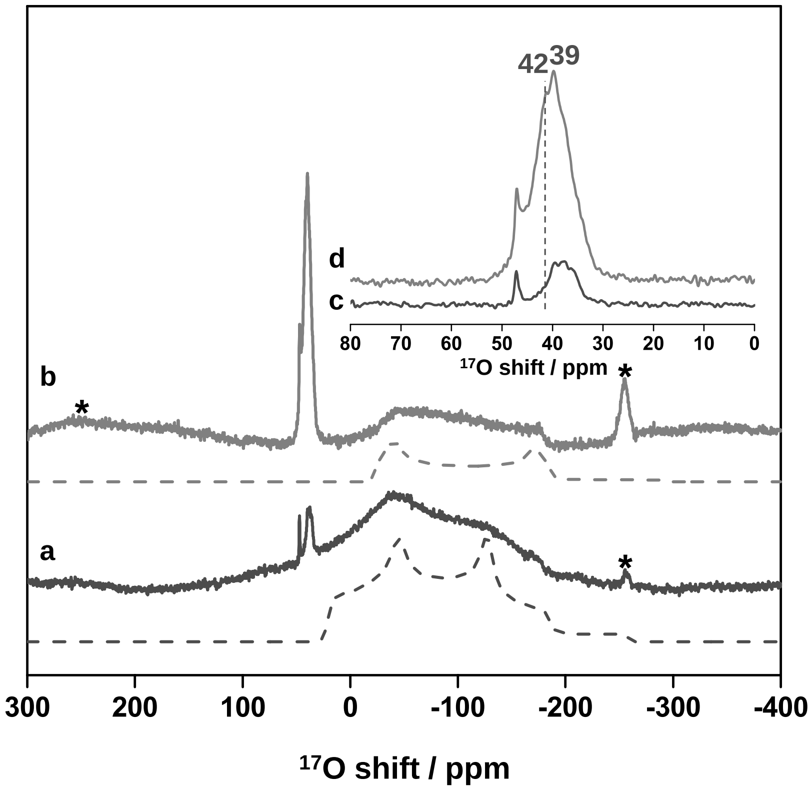

Supplementary Fig. 13. ^{17}O NMR spectrum of non-enriched NS-773 exposed to with $H_2{}^{17}O$ (90%, 16 mbar) (a) and ^{17}O NMR spectrum of the resulting solid heated at 589 K under vacuum (b). External magnetic field: 9.4 T; MAS rate: 16 kHz; recycle delay: 0.5 s. The enlarged regions of from 80 to 0 ppm in (a) and (b) are shown in (c) and (d) (upper right corner). The line shape fitting of the low frequency peaks in (a) and (b) are shown with dashed lines (C_Q = 7.5 MHz, η = 0.4, δ_{iso} = 35 ppm (a) and C_Q = 7.7 MHz, η = 0.1, δ_{iso} = 11 ppm (b)). Asterisks denote spinning sidebands. Both low frequency peaks can be attributed to surface hydroxyls, while the peaks at 47 and 42 to 39 ppm are ascribed to oxygen ions in the bulk and surface, respectively. It is clear that the intensity fraction of the signal at 42 ppm is larger in (b) compared to (a), implying some hydroxyl species are converted to bare surface sites during the heating at 589 K.

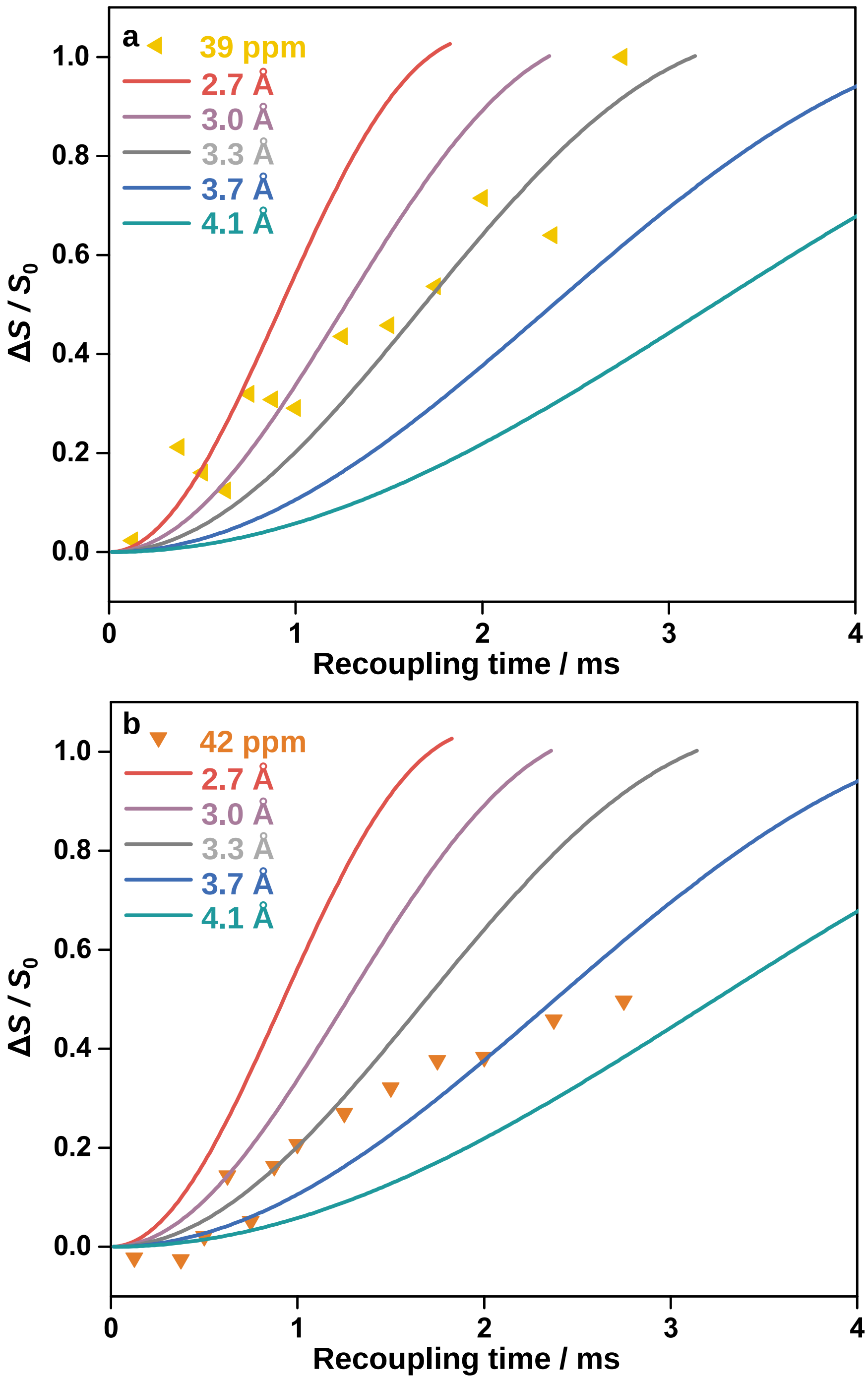

Supplementary Fig. 14. REDOR fraction ($\Delta S/S_0$) for the peak at 39 (a) and 42 ppm (b), respectively, as a function of recoupling time. External magnetic field: 18.8 T; MAS rate: 16 kHz; recycle delay: 5 s. The O-H distance determined for the oxygen species giving rise to the peak at 39 and 42 ppm is approx. 3.0 and 3.7 Å (± 0.3 Å), respectively,

based on a single O-H spin pair simulation. The latter is shorter than the O-H distance of 5.3 Å, assuming this type of oxygen ion is at the fifth coordination shell of an H atom (Figure 3b). Such discrepancy can be understood by the fact that there may be multiple H atoms at this distance (equal or slightly longer than 5.3 Å) away from the specific oxygen ion, making the extracted O-H distance from REDOR NMR shorter.

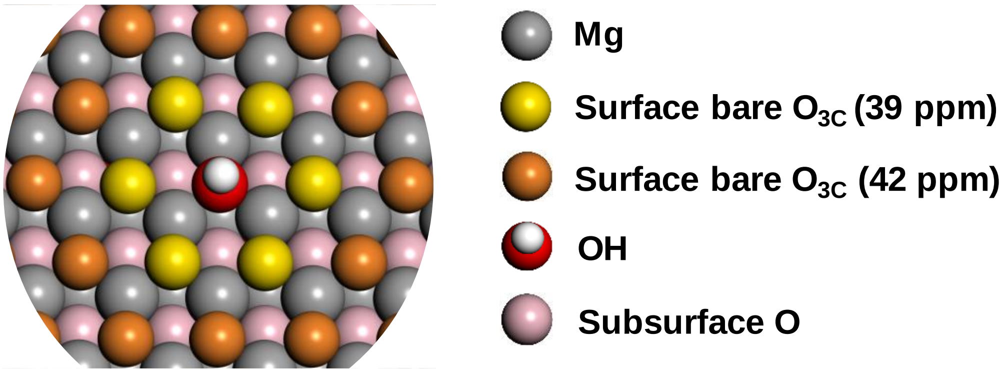

Supplementary Fig. 15. The schematic representation of the surface structure of MgO(111) nanosheets. Both bare oxygen species (O_{3C}) and OH are present. O_{3C} species, which give rise to the ^{17}O NMR resonance at 39 and 42 ppm, are in the third and fifth (or more) coordination shell of H (white) in the OH sites, and are shown in yellow and orange, respectively. H inhibits CO_2 adsorption on O_{3C} sites nearby (39 ppm) while O_{3C} species further away (42 ppm) are not affected and can adsorb CO_2.

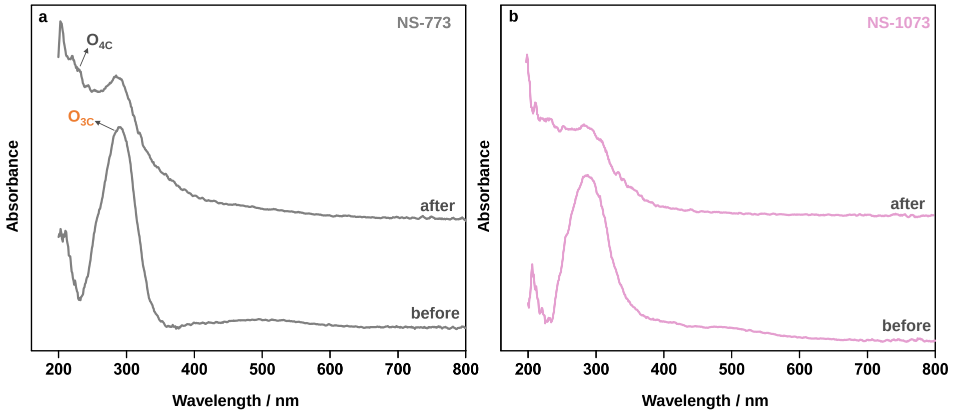

Supplementary Fig. 16. The UV-Vis diffuse reflectance spectra of NS-773 **a** and NS-1073 **b** before and after $^{13}CO_2$ chemisorption.

Density functional theory (DFT) calculations were performed to study CO_2 adsorption on the pristine MgO(111) and hydrogen pre-adsorbed MgO(111) surfaces (Supplementary Fig. 17). The model MgO(111)-4H has four hydrogen atoms being uniformly distributed on the surface and all of the bare surface O_{3C} species are at the third coordination shell of pre-adsorbed hydrogen atoms, while the surface O_{3C} species have no proton at the third coordination in the pristine MgO(111) model (Supplementary Fig. 18). We considered three different configurations of CO_2 adsorption, i.e., the unidentate, bidentate, and tridentate adsorptions. The calculated free energies of CO_2 adsorption (G_{ads}) show that the unidentate adsorption (G_{ads} = -0.81 eV) is more favorable than the bidentate and tridentate adsorptions (G_{ads} = -0.74 and -0.60 eV, respectively) on the pristine MgO(111) surface (see Fig. 4 in the main text and Supplementary Figs. 19-20). However, for the hydrogen pre-adsorbed surface (i.e., MgO(111)-4H), the unidentate and tridentate adsorptions give very similar G_{ads} (-0.27 and -0.32 eV, respectively) and are more favorable than the bidentate adsorption (G_{ads} = -0.11 eV). It is interesting to note that the existence of pre-adsorbed surface hydrogen significantly weakens the interaction of CO_2 with the MgO(111) surface.

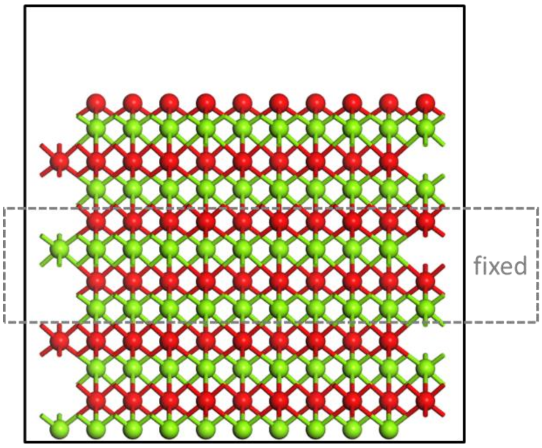

Supplementary Fig. 17. Side view of the 12-layer-slab model of the polar MgO(111). Oxygen and magnesium atoms are in red and green, respectively.

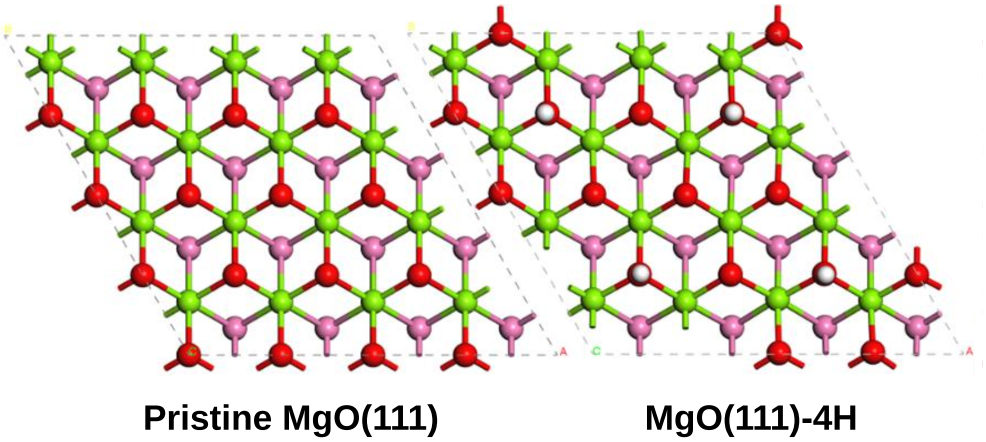

Supplementary Fig. 18. Optimized structures (top view of the top three layers) of the oxygen-terminated pristine MgO(111) and MgO(111)-4H, respectively. Surface oxygen, subsurface oxygen, and magnesium atoms are in red, pink, and green, respectively.

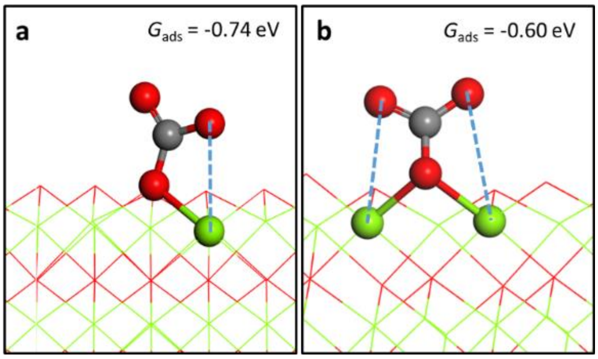

Supplementary Fig. 19. Optimized structures (side view) of the (a) bidentate and (b) tridentate adsorptions of CO_2 at pristine MgO(111). Carbon, oxygen, and magnesium atoms are in grey, red, and green, respectively. Oxygen and magnesium atoms uncoordinated to CO_2 are displayed with lines. The coordination between surface magnesium atoms and CO_2 is expressed with blue dashed lines.

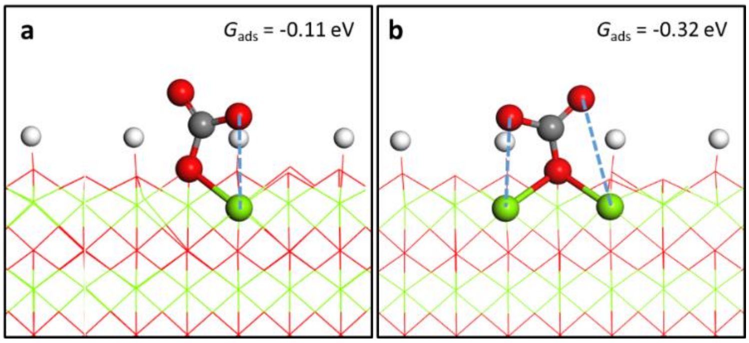

Supplementary Fig. 20. Optimized structures (side view) of the (a) bidentate and (b) tridentate adsorptions of CO_2 at MgO(111)-4H. Hydrogen, carbon, oxygen, and magnesium atoms are in white, grey, red, and green, respectively. Oxygen and magnesium atoms uncoordinated to CO_2 are displayed with lines. The coordination between surface magnesium atoms and CO_2 is expressed with blue dashed lines.

The approach presented in this work can also be extended to study metal/oxide materials, which are widely used as heterogenous catalysts. The ^{17}O NMR spectrum of Au/MgO is compared to the parent MgO nanosheets (NS-1073) in Supplementary Fig. 21. The spectrum of Au/MgO exhibits a broad resonance with contributions from both the peaks at 42 and 39 ppm, along with a sharp component at 47 ppm. The latter corresponds to the oxygen ions in the bulk part of the material, which has almost the same intensity compared to the parent MgO (NS-1073). It is clear that the peak at 42 ppm is associated with a more significant decrease (more than 90%) in intensity than the resonance at 39 ppm (less than around 25%) after Au loading. The decrease in spectral intensity may be ascribed to change of chemical environment due to the presence of Au. Therefore, this preliminary result implies that bare O_{3C} sites on the surface without proton in the 3rd coordination shell may more likely to be the metal binding sites, as compared to the O_{3C} sites with nearby protons.

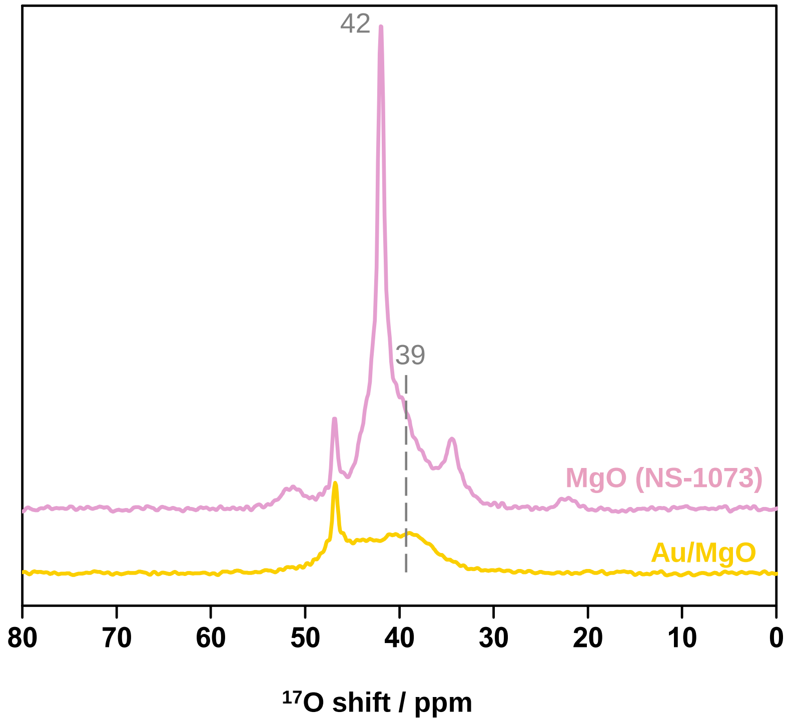

Supplementary Fig. 21. ^{17}O single pulse MAS NMR data of MgO (NS-1073) and corresponding Au/MgO obtained at 9.4 T. MAS rate:16 kHz; recycle delay: 5 s.

Supplementary References

1. Kresse, G. & Furthmuller, J. Efficiency of ab-initio total energy calculations for metals and semiconductors using a plane-wave basis set. *Comput. Mater. Sci.* **6,** 15-50 (1996).

2. Rohrbach, A., Hafner, J. & Kresse, G. Ab initio study of the (0001) surfaces of hematite and chromia: Influence of strong electronic correlations. *Phys. Rev. B* **70,** 125426 (2004).

3. Blöchl, P. E. Projector augmented-wave method. *Phys. Rev. B: Condens. Matter* **50,** 17953-17979 (1994).

4. Perdew, J. P., Burke, K. & Ernzerhof, M. Generalized gradient approximation made simple. *Phys. Rev. Lett.* **77,** 3865-3868 (1996).

5. Speziale, S., Zha, C.-S. & Duffy, T. S. Quasi-hydrostatic compression of magnesium oxide to 52 GPa: Implications for the pressure-volume-temperature equation of state. *J. Geophys. Res-Sol. Ea.* **106,** 515-528 (2001).

6. Chase, M. W. *et al.* JANAF thermochemical tables, 1982 supplement. *J. Phys. Chem. Ref. Data* **11,** 695-940 (1982).
